# Supplementary material for: Pyruvate Dehydrogenase Kinase 1 inhibition mediated oxidative phosphorylation enhancement in cartilage promotes osteoarthritis progression
Source: BMC Musculoskelet Disord. 2023 Jul 20;24:597. doi: 10.1186/s12891-023-06585-6 (PMC10357736; doi:10.1186/s12891-023-06585-6)
Supplement: Supplementary file 1 — Additional file 1. [file 12891_2023_6585_MOESM1_ESM.zip › BP.Gsea.1653623667859/GOBP_CYTOCHROME_COMPLEX_ASSEMBLY.html]

Details for gene set GOBP\_CYTOCHROME\_COMPLEX\_ASSEMBLY[GSEA]

|  || Dataset | 55457\_collapsed\_to\_symbols.cls.cls #OA\_versus\_normal.cls.cls #OA\_versus\_normal\_repos |
| Phenotype | cls.cls#OA\_versus\_normal\_repos |
| Upregulated in class | OA |
| GeneSet | GOBP\_CYTOCHROME\_COMPLEX\_ASSEMBLY |
| Enrichment Score (ES) | 0.63487357 |
| Normalized Enrichment Score (NES) | 1.9630369 |
| Nominal p-value | 0.0023419203 |
| FDR q-value | 0.02940057 |
| FWER p-Value | 0.526 |
Table: GSEA Results Summary

  

Fig 1: Enrichment plot: GOBP\_CYTOCHROME\_COMPLEX\_ASSEMBLY      
 Profile of the Running ES Score & Positions of GeneSet Members on the Rank Ordered List

  

| SYMBOL | TITLE | RANK IN GENE LIST | RANK METRIC SCORE | RUNNING ES | CORE ENRICHMENT || 1 | COX17 | cytochrome c oxidase copper chaperone COX17 [Source:HGNC Symbol;Acc:HGNC:2264] | 64 | 0.966 | 0.1503 | Yes |
| 2 | COA1 | cytochrome c oxidase assembly factor 1 [Source:HGNC Symbol;Acc:HGNC:21868] | 426 | 0.695 | 0.2340 | Yes |
| 3 | COX16 | cytochrome c oxidase assembly factor COX16 [Source:HGNC Symbol;Acc:HGNC:20213] | 498 | 0.660 | 0.3345 | Yes |
| 4 | CYBA | cytochrome b-245 alpha chain [Source:HGNC Symbol;Acc:HGNC:2577] | 501 | 0.658 | 0.4402 | Yes |
| 5 | COA3 | cytochrome c oxidase assembly factor 3 [Source:HGNC Symbol;Acc:HGNC:24990] | 597 | 0.625 | 0.5332 | Yes |
| 6 | TACO1 | translational activator of cytochrome c oxidase I [Source:HGNC Symbol;Acc:HGNC:24316] | 923 | 0.536 | 0.5940 | Yes |
| 7 | SURF1 | SURF1 cytochrome c oxidase assembly factor [Source:HGNC Symbol;Acc:HGNC:11474] | 1829 | 0.381 | 0.5850 | Yes |
| 8 | TTC19 | tetratricopeptide repeat domain 19 [Source:HGNC Symbol;Acc:HGNC:26006] | 2394 | 0.315 | 0.5918 | Yes |
| 9 | FASTKD3 | FAST kinase domains 3 [Source:HGNC Symbol;Acc:HGNC:28758] | 2473 | 0.306 | 0.6349 | Yes |
| 10 | COX15 | cytochrome c oxidase assembly homolog COX15 [Source:HGNC Symbol;Acc:HGNC:2263] | 3727 | 0.183 | 0.5671 | No |
| 11 | HCCS | holocytochrome c synthase [Source:HGNC Symbol;Acc:HGNC:4837] | 4988 | 0.074 | 0.4811 | No |
| 12 | UQCC1 | ubiquinol-cytochrome c reductase complex assembly factor 1 [Source:HGNC Symbol;Acc:HGNC:15891] | 5281 | 0.049 | 0.4664 | No |
| 13 | UQCRFS1 | "ubiquinol-cytochrome c reductase, Rieske iron-sulfur polypeptide 1 [Source:HGNC Symbol;Acc:HGNC:12587]" | 5509 | 0.032 | 0.4539 | No |
| 14 | OXA1L | OXA1L mitochondrial inner membrane protein [Source:HGNC Symbol;Acc:HGNC:8526] | 5974 | -0.003 | 0.4183 | No |
| 15 | COX10 | cytochrome c oxidase assembly factor heme A:farnesyltransferase COX10 [Source:HGNC Symbol;Acc:HGNC:2260] | 6092 | -0.012 | 0.4111 | No |
| 16 | COA4 | cytochrome c oxidase assembly factor 4 homolog [Source:HGNC Symbol;Acc:HGNC:24604] | 7460 | -0.115 | 0.3235 | No |
| 17 | BCS1L | "BCS1 homolog, ubiquinol-cytochrome c reductase complex chaperone [Source:HGNC Symbol;Acc:HGNC:1020]" | 7578 | -0.125 | 0.3345 | No |
| 18 | SCO2 | synthesis of cytochrome C oxidase 2 [Source:HGNC Symbol;Acc:HGNC:10604] | 11410 | -0.488 | 0.1157 | No |
Table: GSEA details [plain text format]

  

Fig 2: GOBP\_CYTOCHROME\_COMPLEX\_ASSEMBLY      
 Blue-Pink O' Gram in the Space of the Analyzed GeneSet

  

Fig 3: GOBP\_CYTOCHROME\_COMPLEX\_ASSEMBLY: Random ES distribution      
 Gene set null distribution of ES for **GOBP\_CYTOCHROME\_COMPLEX\_ASSEMBLY**
